# In this month’s Bulletin

**DOI:** 10.2471/BLT.21.000621

**Published:** 2021-06-01

In this month’s editorial section Adnan A Hyder et al. (406) enumerate the effects of inequitable COVID-19 vaccine distribution. Stefan Listl et al. (407) argue that oral diseases and conditions need to be accounted for in universal health coverage.

In the news section, Andréia Azevedo Soares (410–411) reports on efforts to measure and address psychological distress in health care workers. Gary Humphreys interviews Iman Nuwayhid (412–413) on the rebuilding of public health infrastructure in Beirut, Lebanon.

## African and Caribbean countries 

### Factors affecting diet and physical activity 

Eleanor Turner-Moss et al. (464–472) review evidence for associations with obesity.

## Germany

### Violence against women and children during COVID-19

Cara Ebert & Janina I Steinert (429–438) present prevalence estimates and describe risk factors.

## India

### Methadone dispensing 

Ravindra Rao et al. (422–428) study the logistics of mobile supply.

### Mining health diaries

Neeta Kumar et al. (446–454) examine the utility of data held by individuals.

## Kenya, Uganda and the United Republic of Tanzania 

### Maintaining distance in dense populations 

Joyce Wamoyi et al. (475–476) summarize the difficulties faced by people in informal urban settlements.

## Global

### Violence against children

Yusra Ribhi Shawar & Jeremy Shiffman (414–421) examine the barriers to prioritization of a research agenda.

### Injuries in the kitchen

Joseph S Puthumana et al. (439–445) describe risk factors for burn injuries in children.

### Misinformation on social media

Elia Gabarron et al. (455–463) review the extent of misleading information related to COVID-19.

### Collateral effects of pandemic control measures

Jared M Alswang et al. (473–474) argue for the maintenance of neglected tropical disease programmes.

